# The Influence of the Ventricular-Lumbar Gradient on Cerebrospinal Fluid Analysis in Serial Samples

**DOI:** 10.3390/brainsci12030410

**Published:** 2022-03-20

**Authors:** Franz Felix Konen, Peter Lange, Ulrich Wurster, Konstantin Fritz Jendretzky, Stefan Gingele, Nora Möhn, Kurt-Wolfram Sühs, Martin Stangel, Thomas Skripuletz, Philipp Schwenkenbecher

**Affiliations:** 1 Clinical Neuroimmunology and Neurochemistry, Department of Neurology, Hannover Medical School, 30625 Hannover, Germany; konen.felix@mh-hannover.de (F.F.K.); wurster.ulrich@mh-hannover.de (U.W.); jendretzky.konstantin@mh-hannover.de (K.F.J.); gingele.stefan@mh-hannover.de (S.G.); moehn.nora@mh-hannover.de (N.M.); suehs.kurt-wolfram@mh-hannover.de (K.-W.S.); stangel.martin@mh-hannover.de (M.S.); skripuletz.thomas@mh-hannover.de (T.S.); 2 Department of Neurology, University Medical Center Göttingen (UMG), 37075 Göttingen, Germany; peter-la@med.uni-goettingen.de

**Keywords:** cerebrospinal fluid, immunoglobulins, virus-specific antibody index, ventriculo-lumbar gradient, idiopathic intrathecal hypertension, normal pressure hydrocephalus

## Abstract

Background: Cerebrospinal fluid (CSF) samples from patients with non-inflammatory neurological diseases are used for control groups in biomarker studies. Since large amounts of CSF are withdrawn, patients with idiopathic intracranial hypertension (IIH) or normal pressure hydrocephalus (NPH) are especially suitable. The serially taken CSF portions are usually collected in different tubes. We aimed to investigate whether the later random choice of one of these tubes for CSF investigations might harbor the risk of different CSF protein findings due to the so-called ventriculo-lumbar CSF gradient. Methods: Patients with IIH (9) and NPH (7) were included. CSF was serially taken and collected in six tubes of 5 mL each. Concentrations and CSF-serum quotients of immunoglobulins, albumin and the virus-specific antibody index (AI) were determined in the first, fourth and sixth CSF fraction. Results: CSF immunoglobulin and albumin concentrations and CSF-serum protein quotients were significantly lower in the fourth and sixth CSF fraction compared with the first CSF fraction. Virus-specific AI did not significantly differ in the different CSF fractions. Conclusions: CSF protein analytics should be performed in the first CSF fraction in order to avoid different measurement results and achieve comparability within a control group and between different control and patient groups.

## 1. Introduction

The choice of an appropriate control group is essential in studies investigating biomarkers in cerebrospinal fluid (CSF) [1]. Patients with idiopathic intrathecal hypertension (IIH) or normal pressure hydrocephalus (NPH) are especially suitable for the control group of non-inflammatory neurological disease (NIND), due to the large amount of CSF that is obtained during routine lumbar puncture (LP) [1,2,3,4,5,6]. In both diseases, only a small fraction of the CSF sample is used for diagnostic purpose, while the majority of the sample from the therapeutic CSF drainage remains as leftover [7].

The total volume of the CSF compartment in adults compromises approximately 140 mL, and many CSF proteins are not homogenously distributed within the CSF compartment [8,9,10]. Concentrations of blood-derived proteins, such as albumin, are higher in the lumbar than in the cranial region, which is called the ventriculo-lumbar CSF gradient [10,11,12,13]. On the other hand, some neuronal damage markers originate in the brain and therefore have higher concentrations in the cranial than in the lumbar region [10,11,12,13].

When large amounts of CSF are taken during one LP, usually several serially fractions of CSF are collected in different sample tubes. When these CSF samples are stored for later use in control groups, there is usually no mention of which portion of the CSF aliquot was used [1]. We hypothesized that the random selection of serially taken CSF samples from one patient can result in significantly different CSF protein findings among the aliquots, even though all aliquots originate from the same patient.

In order to evaluate the influence of the ventriculo-lumbar CSF gradient, we determined the CSF protein concentration, the CSF–blood-barrier function, the intrathecal synthesis of immunoglobulins and the virus-specific intrathecal antibody synthesis in serially taken CSF samples in a cohort of patients with IIH and NPH.

## 2. Materials and Methods

### 2.1. Patients

CSF and serum samples were obtained from patients with IIH (*n* = 9) and NPH (*n* = 7) who were treated in Hannover Medical School between 2018 and 2019. All patients received LP for standard diagnostic or treatment purpose. After the spinal needle was inserted between the lumbar vertebrae L3/L4, L4/L5 or L5/S1, 6 CSF fractions, each with 5 mL CSF, were serially taken during the LP (fraction 1, from start to 5th mL CSF; fraction 2, from 5th to 10th mL CSF; fraction 3, from 10th to 15th mL CSF; fraction 4, from 15th to 20th mL CSF; fraction 5, from 20th to 25th mL CSF; fraction 6, from 25th to 30th mL CSF). LP was performed in sitting position in patients with NPH and in lying position in patients with IIH. A blood sample was taken instantly after LP from each patient.

### 2.2. CSF and Serum Analytical Procedures

In the first CSF fraction, CSF cells were counted microscopically with Fuchs Rosenthal counting chamber (normal CSF cell count was defined <5 cells/µL). CSF samples with elevated cell count were not included. CSF protein measurements were performed in the first, fourth and sixth CSF fraction. CSF total protein (cutoff = 500 mg/L) was measured by using a Bradford dye-binding procedure [14]. Concentrations of albumin, immunoglobulin G (IgG), IgA and IgM were determined by kinetic nephelometry (Beckman Coulter IMMAGE, Brea, CA, USA) and corresponding serum sample. CSF-serum quotients were calculated for albumin (Q Albumin), IgG (Q IgG), IgA (Q IgA) and IgM (Q IgM). The IgG index is defined as the quotient of CSF IgG/CSF albumin and serum IgG/serum albumin. CSF-serum albumin quotients (Q Albumin) were applied to assess the blood–CSF barrier function. The age-adjusted upper reference limit was calculated according to the following formula: 8 + (age in years/25) [15]. Quantitative intrathecal synthesis of IgG, IgA and IgM was determined by using the method of Reiber–Felgenhauer [14]. CSF-specific oligoclonal bands (OCB) were determined by isoelectric focusing on polyacrylamide gels (EDC, Tübingen, Germany) with consecutive silver staining [16].

The intrathecally produced antibodies against the viruses measles, rubella, varicella zoster (VZV) and herpes simplex virus (HSV) were determined by calculating the virus-specific antibody index (AI) according to the following formula: (CSF virus-IgG/serum virus-IgG)/(CSF IgG total/serum IgG total). In the case of a detectable intrathecal total-IgG production by the method of Reiber–Felgenhauer, the calculation of a corrected AI according to Reiber and Lange is generally recommended [17,18,19]. AI ≥ 1.5 was considered to be elevated [17,18,19].

Virus-IgG was measured in CSF and serum by ELISA kits from Virion/Serion (Würzburg, Germany). To improve sensitivity, the detection antibody from the ELISA kit was exchanged for a polyclonal rabbit anti-human IgG-horseradish peroxidase (HRP) from Agilent (Santa Clara, CA, USA). CSF and serum sample concentrations of virus-IgG were determined in reference to the standard curve [17].

The Neurochemistry Laboratory of the Department of Neurology participates regularly in the external INSTAND survey program for analytic methods quality control [20].

### 2.3. Statistical Analysis

For statistical analysis, GraphPad Prism (La Jolla, CA, USA; version 5.02) was used. Values in the main text are presented as median and range. Values in figures and Supplementary figures are depicted as median, minimum and maximum if not described otherwise. The Shapiro–Wilk normality test was used to asses for normal distribution of values. Mann–Whitney U test was used for independent values. Kruskal–Wallis test (independent values) and Friedman test (dependent values) with Dunn’s Multiple Comparison post hoc test was used for group comparison and Wilcoxon signed rank test for comparison of two groups with repeated measurements. Univariable linear regression analyses were performed, with CSF fractions (*x*-axis) representing the independent value and albumin as well as immunoglobulin CSF concentrations (*y*-axis) being the dependent values. The *p*-values < 0.05 were determined to be statistically significant.

## 3. Results

### 3.1. Differences between Serially Taken CSF Fractions in Patients with NIND

Of the 16 patients included, nine patients were diagnosed with IIH (seven women, median age 30 years; range, 18–64 years) and seven patients were diagnosed with NPH (three women, median age 78 years; range, 55–84 years).

**Figure 1:**
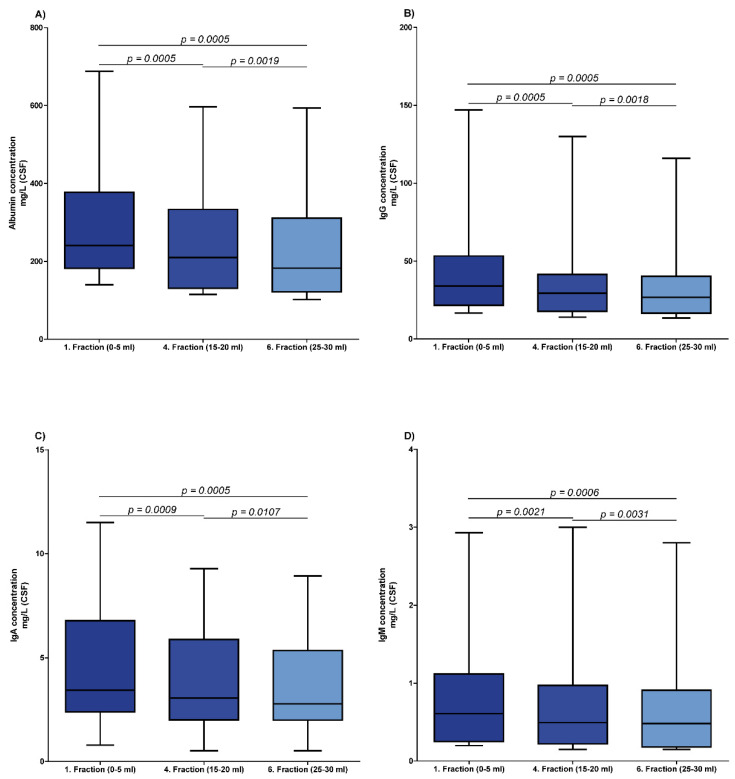
Cerebrospinal fluid (CSF) protein concentrations in different CSF fractions. Depicted are CSF concentrations of albumin (**A**), IgG (**B**), IgA (**C**) and IgM (**D**) of all included patients (*n* = 16) suffering from normal pressure hydrocephalus (NPH) and idiopathic intracranial hypertension (IIH) in three different CSF fractions. A *p*-value indicating the level of statistical significance is depicted above the line.

Figure 1 displays the results of the serially taken CSF fractions (CSF fractions 1, 4 and 6). The median CSF concentrations of albumin, IgG, IgA and IgM were significantly lower in the fourth (albumin, 210 mg/L; IgG, 29.35 mg/L; IgA, 3.065 mg/L; and IgM, 0.4935 mg/L) and the sixth (albumin, 182.5 mg/L; IgG, 26.8 mg/L; IgA, 2.78 mg/L; and IgM, 0.4810 mg/L) CSF fraction as compared to the first (albumin, 240.5 mg/L; IgG, 33.95 mg/L; IgA, 3.44 mg/L; and IgM, 0.609 mg/L) CSF fraction. The most significant difference was found between the first and fourth CSF fraction for albumin, IgG, IgA and IgM (*p*-values are displayed in Figure 1). In addition, the quotients of albumin, IgG, IgA and IgM were significantly lower in the fourth (Q Albumin, 5.51; Q IgG, 2.837; Q IgA, 1.343; Q IgM, 0.4497) and sixth (Q Albumin, 4.862; Q IgG, 2.595; Q IgA, 1.151; Q IgM, 0.4384) CSF fractions than in the first (Q Albumin, 6.154; Q IgG, 3.292; Q IgA, 1.424; and Q IgM, 0.5592) CSF fraction. The *p*-values for the comparison of CSF-serum concentration quotients are displayed in Supplementary Materials Figure S1. However, a statistically significant linear regression was not found (*p*-values between 0.0763 and 0.1218). The median IgG index also did not differ significantly among the different fractions (first CSF fraction, 0.5041; fourth CSF fraction, 0.5142; sixth CSF fraction, 0.5116; *p*-values: 0.7761 and 0.1150), as depicted in Supplementary Materials Figure S2. The median IgA and IgM index did not differ significantly in the fourth (IgA index, 0.2448; and IgM index, 0.0984) and sixth (IgA index, 0.2509; and IgM index, 0.1145) CSF fraction compared with the first (IgA index, 0.2537, and IgM index, 0.1253) CSF fraction (*p*-values between 0.3531 and 0.6545; Supplementary Materials Figure S2).

An intrathecal immunoglobulin synthesis calculated with Reiber’s diagram could not be detected in any of the CSF samples.

**Figure 2:**
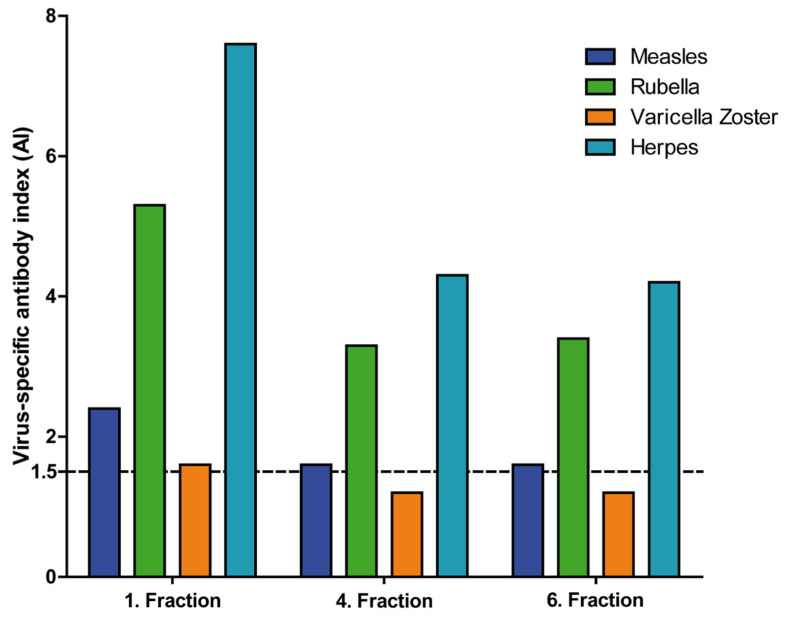
Virus-specific antibody indices (AI) in a patient suffering from multiple sclerosis and normal pressure hydrocephalus (NPH). Depicted are the virus-specific AI in three different cerebrospinal fluid (CSF) fractions in a measle-, rubella-, varicella zoster virus (MRZ)-positive patient suffering from secondary progressive multiple sclerosis (SPMS) and NPH. The first CSF fraction consisted of the first 5 mL obtained at the lumbar puncture, the fourth fraction of the 15–20 mL and the sixth fraction of the 25–30 mL. Shown are the AIs for measle virus, rubella virus, varicella zoster virus and herpes simplex virus. The dashed line at 1.5 represents the cutoff for positive virus-specific AI.

Virus-specific AIs were below the cutoff in all but one patient in the first (medians between 0.9 and 1.0) CSF fraction, and values did not significantly differ in the fourth (medians between 0.9 and 1.0) and sixth (medians between 0.9 and 1.0) CSF fractions (*p* = 0.3182). The one patient with elevated virus-specific AI (measles, rubella, VZV and HSV) was diagnosed with secondary progressive multiple sclerosis and NPH. Virus-specific AI were lower in the fourth and sixth CSF fraction in this patient. Details are depicted in Figure 2. The AI against VZV even decreased under the cutoff of 1.5, indicating a normal AI in the fourth and sixth CSF fraction.

### 3.2. Differences in the First CSF Fraction between IIH and NPH

Details of protein findings in the first CSF fraction in IIH and NPH are shown in Supplementary Materials Figure S3. Median concentrations of the CSF proteins albumin, IgG, IgA and IgM in the first fraction were not significantly different in patients with IIH or NPH. Similar results were found for the median quotients of albumin, IgG, IgA and IgM, which did not differ significantly between IIH and NPH.

A quantitative intrathecal immunoglobulin synthesis was not found in any patient, whereas OCB restricted to CSF were found in two patients, one patient with IIH and the other patient with NPH, who suffered from additional multiple sclerosis.

## 4. Discussion

This study demonstrates that CSF protein concentrations are significantly higher in the first CSF fractions than in following CSF fractions during LP with serially taken CSF samples. This observation includes both the solely blood-derived protein albumin and immunoglobulins of all subclasses, which are blood-derived and intrathecal-derived.

Blood-derived proteins diffuse into the CSF compartment along the passage from secretion in the ventricles to spine and reabsorption, while brain-derived proteins have an efflux from the CSF into the blood [21,22,23,24]. Consequently, the composition of CSF alters during its passage through the neuroaxis, and the CSF flow decelerates from ventricular to lumbar, which results in the ventriculo-lumbar CSF gradient [25]. This leads to a linear increase of concentrations of blood-derived proteins and a non-linear decrease of brain-derived proteins under physiological conditions [24,26,27]. We could confirm this hypothesis by demonstrating that the albumin concentration and also the CSF-serum albumin quotient is significantly lower in the fourth and sixth CSF fraction. This harbors the risk to overlook a blood-CSF barrier dysfunction when not the first CSF fraction is analyzed. In a previous study, significant gradients for albumin and the CSF-serum albumin quotients were found, but significant differences between the first and the last CSF fractions could not be detected [28]. In another study, median albumin concentrations were reported to be 1.25-to-1.5-fold higher in the first 5 mL fraction than in the eighth 5 mL fraction, which is in line with our observations [11,12,13]. However, despite the significant differences of immunoglobulin and albumin concentrations between the serially taken CSF samples, a significant linear regression for the decrease of concentrations could not have been found in the present study. The most likely explanation might be that the concentration differences between the fourth and the sixth CSF fraction were not as distinctive as between the first and the fourth CSF fraction, which might result in a non-significant linear regression.

Immunoglobulins in CSF can either be exclusively blood-derived or a combination of blood-derived and synthesized within the CSF compartment. In our study, an intrathecal immunoglobulin synthesis using Reiber’s diagram was not found in any patient, and virus-specific AI were below the cutoff in all but one patient, indicating a blood-derived origin. The one patient with elevated virus-specific AI suffered from NPH and multiple sclerosis. Since the CSF immunoglobulin concentrations and the quotients were significantly lower in the fourth and sixth CSF fraction than in the first CSF fraction, we conclude that the ventriculo-lumbar CSF gradient also applies for CSF concentrations of blood-derived immunoglobulins [9,10]. In two other studies, the IgG index, which is calculated by dividing the immunoglobulin G quotient by the albumin quotient, did not differ significantly in successive CSF portions, which is in agreement with our findings [28,29].

On the other hand, we can only speculate about the distribution of intrathecally synthesized immunoglobulins in the CSF compartment, since in none of the patients was an intrathecal synthesis detected by Reiber’s diagram. In one study, an increase of the IgG index in successive portions of CSF in patients with multiple sclerosis has been described [30]. Although none of our patients showed an intrathecal immunoglobulin synthesis, one patient with virus-specific AI in the first CSF fraction might, however, serve as a model for intrathecally synthesized immunoglobulins. In this patient, AI were lower in in the fourth and sixth CSF fraction. The AI against VZV was even below the cutoff in the following CSF fraction. However, AI include Q IgG and thus are thought to possibly normalize differences between CSF fractions. Nevertheless, we could demonstrate that the intrathecally produced virus-specific IgG fraction had a lower concentration in the fourth and sixth fraction in the one patient suffering from NPH and multiple sclerosis. Since the distribution of intrathecally synthesized IgG is not known, we hypothesized that the decrease of the intrathecally produced virus-specific IgG fraction is more pronounced compared to the IgG concentration. We therefore speculate that the ventriculo-lumbar CSF gradient affects concentrations of intrathecally synthesized immunoglobulins more than blood-derived immunoglobulins, hazarding false-negative AI and misdiagnosis of CNS infections when not the first 5mL are taken for CSF analyses.

On the other hand, it might be speculated that the results of the CSF analysis of the first fraction are false negative, while the results of the last CSF fraction are actually correct. Following this, one might consider to rather choose among different CSF fractions the one less influenced by blood-driven proteins, such as the sixth fraction, in order to determine intrathecally synthesized antibodies. However, further investigation of this finding might be difficult, since an intrathecal immunoglobulin synthesis is associated with autoimmune or infectious diseases in which usually lower amounts of CSF are taken for diagnostic purposes, while removal of more CSF is associated with higher rates of side effects, such as headache. Furthermore, an analysis of the first fraction of CSF is considered as standard for diagnostic purposes.

Another observation of our study is that CSF concentrations and quotients of albumin and immunoglobulins in the first CSF fraction were not significantly different between IIH and NPH patients. Although NPH patients were found to be significantly older than IIH patients (*p* = 0.0014), significant differences concerning the CSF albumin concentrations or the age-dependent Q Albumin were not observed [15,24]. Both disease entities are subsumed as NIND according to Teunissen et al. and might therefore be equivalently used as controls in CSF biomarker studies [1].

## 5. Conclusions

The choice of which CSF fraction from serially taken CSF samples is used can have a significant impact on CSF concentrations of albumin, IgG, IgA and IgM, as well as the respective CSF-serum concentration quotients. We therefore suggest using the first (5 mL) CSF fraction for analytics to improve diagnostic validity. If the first CSF fraction is not used, then we recommend to state which CSF fraction was used.

## Data Availability

Data supporting the findings can be found in the tables. Additional data extracted may be shared upon request.

## Supplementary Materials

The following supporting information can be downloaded at: https://www.mdpi.com/article/10.3390/brainsci12030410/s1, Figure S1: Cerebrospinal fluid (CSF)-serum concentration quotients in different CSF fractions. Depicted are cerebrospinal fluid (CSF)-serum concentration quotients of albumin (Q Albumin, A), IgG (Q IgG, B), IgA (Q IgA, C) and IgM (Q IgM, D), as well as the IgG index in normal pressure hydrocephalus (NPH) and idiopathic intracranial hypertension (IIH) patients in three different CSF fractions. Figure S2: Immunoglobulin indices in different cerebrospinal fluid (CSF) fractions. Depicted are the IgG, IgA and IgM index in normal pressure hydrocephalus (NPH) and idiopathic intracranial hypertension (IIH) patients in three different CSF fractions. Indices of all immunoglobulins were not significantly different between the first and the fourth or the fourth and the sixth CSF fraction. Values are presented as median with interquartile range. Figure S3: Cerebrospinal fluid (CSF) concentrations in patients with idiopathic intrathecal hypertension (IIH) and normal pressure hydrocephalus (NPH). Depicted are cerebrospinal fluid (CSF) concentrations of albumin (A), IgG (B), IgA (C) and IgM (D) in normal pressure hydrocephalus (NPH) and idiopathic intracranial hypertension (IIH) patients in first CSF fraction. Above the line, *p*-value indicating the level of statistical significance is depicted.
