# Supplementary figures and images for: The Influence of the Ventricular-Lumbar Gradient on Cerebrospinal Fluid Analysis in Serial Samples
Source: Brain Sci. 2022 Mar 20;12(3):410. doi: 10.3390/brainsci12030410 (PMC8946585; doi:10.3390/brainsci12030410)

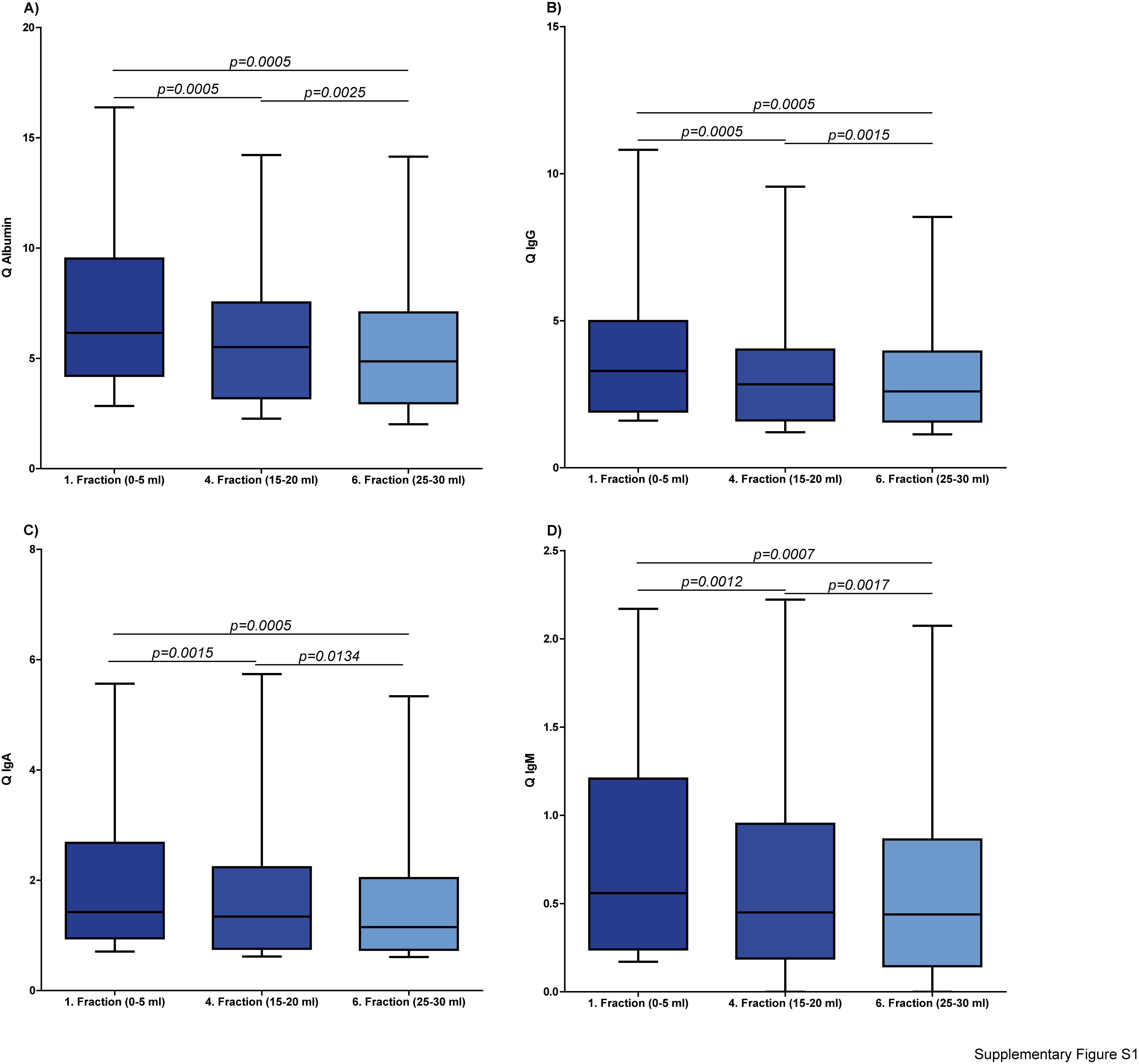

Supplement: Supplementary file 1 [file brainsci-12-00410-s001.zip › Supplementary Figure S1.tif]

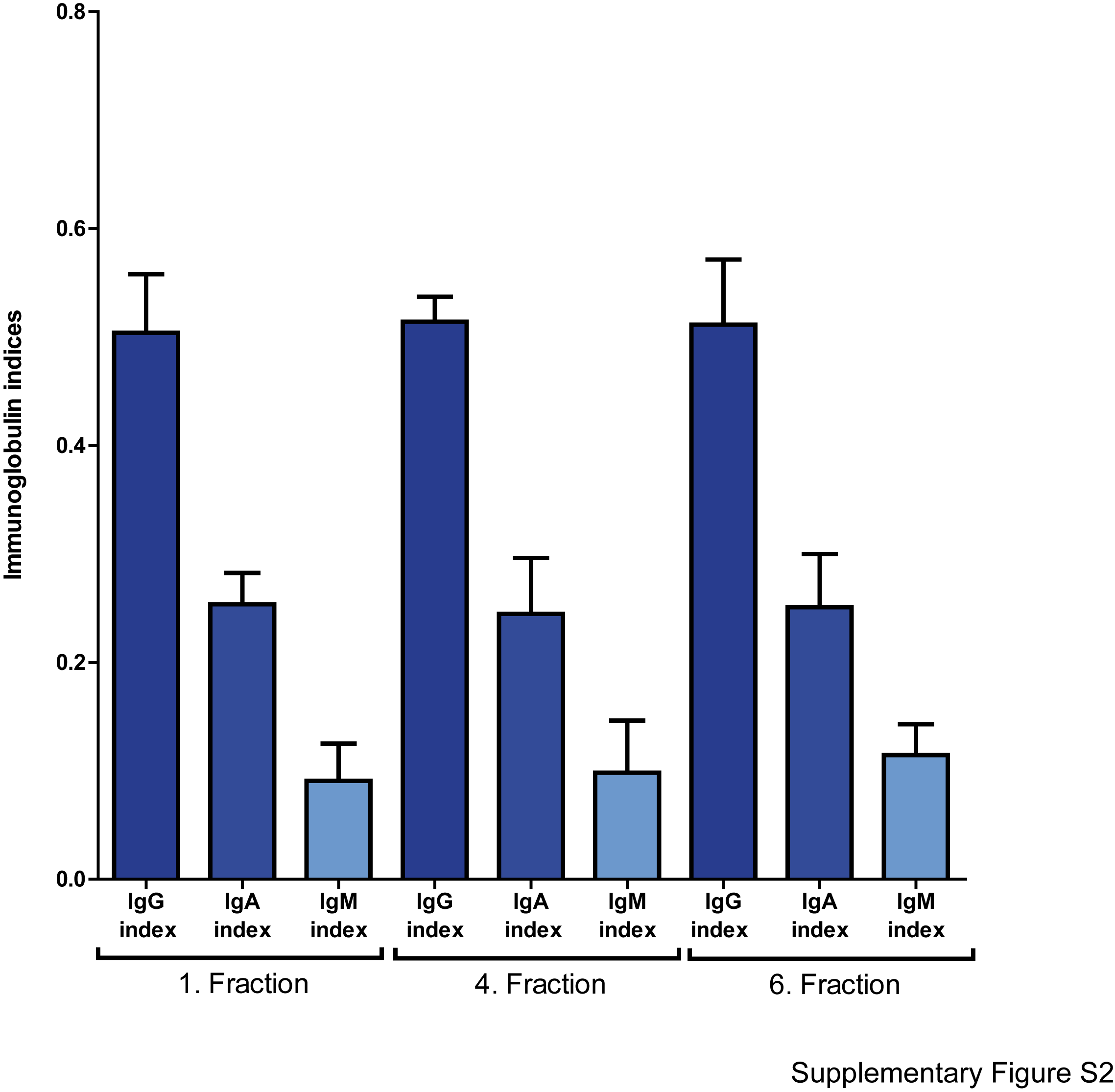

Supplement: Supplementary file 1 [file brainsci-12-00410-s001.zip › Supplementary Figure S2.tif]

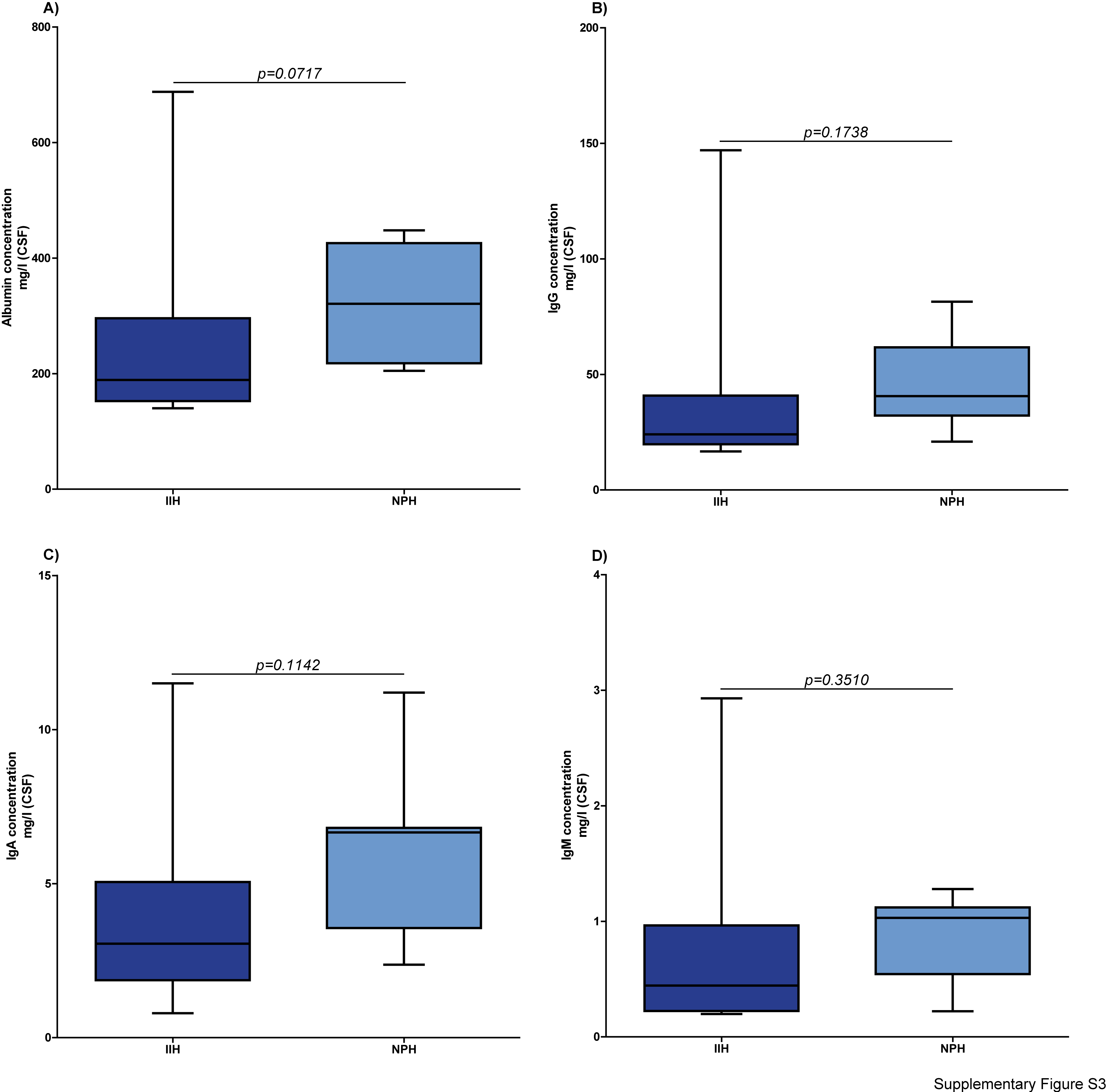

Supplement: Supplementary file 1 [file brainsci-12-00410-s001.zip › Supplementary figure S3.tif]
